# Risk Phenotyping Before Graft Implantation: FTIR Spectroscopy and Machine Learning for Complementary Risk Stratification in Kidney Transplantation

**DOI:** 10.3390/medsci14030353

**Published:** 2026-06-27

**Authors:** Luis Ramalhete, Rúben Araújo, Emanuel Vigia, Miguel Bigotte Vieira, Anibal Ferreira, Cecilia R. C. Calado

**Affiliations:** 1 Blood and Transplantation Center of Lisbon, Instituto Português do Sangue e da Transplantação, Alameda das Linhas de Torres, No. 117, 1769-001 Lisbon, Portugal; 2 NOVA Medical School, Faculdade de Ciências Médicas, Universidade NOVA de Lisboa, 1169-056 Lisbon, Portugal; 3 iNOVA4Health—Advancing Precision Medicine, Núcleo de Investigação em Doenças Renais, NOVA Medical School, Faculdade de Ciências Médicas, Universidade NOVA de Lisboa, 1169-056 Lisbon, Portugal; 4 LBS—Lisbon Business & Government School, Rua de São Bernardo, 34-A, 1200-427 Lisbon, Portugal; 5 Hepatobiliopancreatic and Transplantation Center, Curry Cabral Hospital, Unidade Local de Saúde de São José, R. da Beneficência 8, 1050-099 Lisbon, Portugal; 6 Centro Clínico Académico de Lisboa, Faculdade de Ciências Médicas, Universidade NOVA de Lisboa, 1169-056 Lisbon, Portugal; 7 Nephrology, Hospital Curry Cabral, Unidade Local de Saúde de São José, R. da Beneficência 8, 1050-099 Lisbon, Portugal; 8 ISEL—Instituto Superior de Engenharia de Lisboa, Instituto Politécnico de Lisboa, Rua Conselheiro Emídio Navarro 1, 1959-007 Lisbon, Portugal; 9 Institute for Bioengineering and Biosciences (iBB), The Associate Laboratory Institute for Health and Bioeconomy-i4HB, Instituto Superior Técnico (IST), Universidade de Lisboa (UL), Av. Rovisco Pais, 1049-001 Lisbon, Portugal

**Keywords:** kidney transplantation, rejection, FTIR spectroscopy, serum, machine learning

## Abstract

Background: Rejection remains a major barrier to long-term kidney allograft survival, and pre-transplant risk stratification remains incomplete. This study evaluated whether pre-transplant serum Fourier-transform infrared (FTIR) spectra, analyzed using machine learning methods, could identify kidney transplant recipients at increased risk of subsequent biopsy-proven rejection. Methods: In this retrospective single-center study, 80 pre-transplant serum samples collected on the day of transplantation were initially evaluated; after spectral quality control, 79 samples were retained for analysis. FTIR spectra were acquired in transmission mode and analyzed in the 600–1900 cm^−1^ and 2800–3400 cm^−1^ regions. Multiple preprocessing strategies were assessed, including Rubber Band baseline correction, vector normalization, and first- and second-derivative transformation, with and without normalization. Naïve Bayes classifiers with Leave-One-Out Cross-Validation and Fast Correlation-Based Filter feature selection were applied. Results: Exploratory analysis showed broad overlap between groups, indicating a subtle multivariate spectral signal. In the initial exploratory workflow, classifier performance depended strongly on preprocessing and feature selection. Because non-nested feature selection may produce optimistic estimates, the main supervised analysis was repeated using FCBF nested within each LOOCV training fold. The best-performing nested model was obtained using second derivative transformation followed by normalization in the combined 600–1900 and 2800–3400 cm^−1^ regions, achieving an AUC of 0.837, accuracy of 0.747, sensitivity of 0.675, specificity of 0.821, balanced accuracy of 0.748, and F1-score of 0.730. Permutation testing with 1000 label-randomized repetitions supported performance above chance expectation, with no permuted model reaching the observed AUC (empirical *p* = 0.000999). Conclusions: Pre-transplant serum FTIR spectroscopy combined with leakage-aware nested machine learning analysis identified an internally validated spectral signal associated with subsequent biopsy-proven rejection. These findings support FTIR as a promising complementary and hypothesis-generating approach for pre-transplant biochemical risk phenotyping, requiring external multicenter validation before clinical application.

## 1. Introduction

Kidney transplantation is the preferred treatment for end-stage kidney disease, yet long-term graft survival remains limited by alloimmune injury, particularly rejection. In contemporary practice, rejection is still fundamentally defined and categorized through biopsy-based assessment within the Banff framework. At the same time, the Banff 2022 Kidney Meeting Report and subsequent molecular pathology work make clear that rejection phenotyping remains an evolving field, particularly with respect to antibody-mediated injury, microvascular inflammation, and the integration of transcript-based biopsy diagnostics. Accordingly, current rejection assessment is both indispensable and still biologically incomplete [1,2].

Although biopsy remains the reference standard, it is not an ideal stand-alone strategy for repeated surveillance or earlier biological risk stratification. It is invasive, subject to sampling limitations and interpretive variability, and carries a measurable complication burden. In a recent national prospective cohort, major complications after transplant kidney biopsy occurred in 1.4% of procedures. These limitations have sustained interest in adjunctive biomarkers that might refine the context in which biopsy is used, rather than replace histology itself [3,4,5,6].

Before transplantation, immunologic risk assessment already relies on several well-established variables, including sensitization history, calculated or panel-reactive antibodies, donor-specific anti-HLA antibodies (DSA), and donor–recipient human leukocyte antigen (HLA) compatibility. These metrics are highly relevant, particularly in sensitized recipients, and preformed donor-specific antibodies remain strongly associated with antibody-mediated rejection and inferior graft outcomes. Likewise, increasing HLA mismatch burden and prior sensitizing events such as transfusion, pregnancy, or previous transplantation remain central to risk assessment. However, these variables still do not fully resolve which recipients will later develop biopsy-proven rejection [7,8,9,10,11,12,13,14,15,16,17].

Over the last few years, the field of non-invasive biomarker development in kidney transplantation has expanded substantially. Among circulating biomarkers, donor-derived cell-free DNA is one of the best-validated candidates and, in a large 2024 population-based study, improved the detection of kidney allograft rejection beyond standard-of-care monitoring. However, broader translation remains incomplete. The prospective multicenter EU-TRAIN study illustrated this clearly: although multiple blood biomarkers were evaluated, circulating anti-HLA donor-specific antibodies retained the clearest association with rejection, whereas several other candidate markers showed limited added value beyond standard monitoring. Parallel work on blood gene-expression profiling, urinary chemokines, extracellular vesicles, and molecular gene-panel approaches supports the biological promise of non-invasive monitoring, but also highlights continuing issues of context of use, reproducibility, and clinical integration [18,19,20,21,22,23,24,25,26,27,28,29,30].

In this context, Fourier-transform infrared spectroscopy offers a conceptually distinct and potentially complementary strategy. Rather than quantifying a single analyte, FTIR captures a global biochemical fingerprint of the sample, enabling rapid, label-free, low-volume analysis that can be coupled with machine-learning methods for classification and risk modeling. The translational appeal of biofluid FTIR lies precisely in this systems-level readout, but its clinical development also depends on rigorous pre-analytical control, standardized workflows, and validation across realistic datasets [31,32,33,34,35,36,37,38,39,40,41,42].

Our group has recently contributed to this area at several complementary levels. We reviewed the broader landscape of rejection-focused precision medicine in kidney transplantation and showed that serum FTIR signatures combined with machine learning could discriminate kidney allograft rejection, while serum molecular fingerprinting was also associated with cellular rejection. We further showed that transplant-associated biofluids other than serum can yield clinically informative FTIR patterns; taken together, these findings support the biological plausibility of spectroscopic risk modeling in transplantation and indicate that the platform should be positioned as complementary rather than a replacement for established approaches [5,43,44,45,46,47].

Crucially, a pre-transplant serum approach should not be framed as detecting rejection before it exists. Rather, its potential value lies in identifying a baseline serum spectral biochemical signature associated with increased susceptibility to subsequent rejection after graft implantation. This distinction is important both biologically and clinically. Against this background, the present study aimed to evaluate whether pre-transplant serum FTIR spectra, analyzed using machine-learning methods, could identify kidney transplant recipients at increased risk of subsequent biopsy-proven rejection.

## 2. Materials and Methods

### 2.1. Study Population and Baseline Characteristics

This retrospective observational single-center study was conducted at Unidade Local de Saude de São José (ULS São José), Hospital Curry Cabral. The study was approved by the ULS São José Ethics Committee (approval no. 1215/2022), and all patients provided written informed consent.

Adult kidney transplant recipients with an available pre-transplant serum sample collected on the day of transplantation, before graft implantation, were eligible for inclusion. Patients younger than 18 years and patients with HIV infection were excluded. A total of 80 pre-transplant serum samples were initially evaluated. After FTIR spectral quality control, one spectrum was excluded, resulting in a final analytical cohort of 79 patients.

The primary outcome was biopsy-proven rejection after kidney transplantation during post-transplant follow-up. Rejection episodes were further categorized descriptively as humoral, cellular, or mixed rejection when this information was available. Subtype-specific machine-learning modelling was not performed because the number of events within each rejection subgroup was limited. Baseline demographic, clinical, and immunologic variables were collected to characterize the study cohort and are summarized in Table 1. These variables included age at transplantation, sex, dialysis modality, retransplantation status, donor type, PRA-CDC max, pre-transplant anti-HLA antibodies, pre-transplant donor-specific antibodies, and the total number of HLA mismatches, including HLA-A, HLA-B, and HLA-DR mismatches. Post-transplant immunosuppression was managed according to the institutional standard-of-care practice of the kidney transplantation unit. In line with published experience from the same center, induction immunosuppression was risk-adapted: higher immunological- or donor-related risk recipients were generally treated with thymoglobulin-based induction, whereas lower-risk recipients received basiliximab. Highly sensitized recipients could also receive intravenous immunoglobulin when clinically indicated. All recipients received perioperative corticosteroid induction, and maintenance immunosuppression was generally based on tacrolimus, mycophenolate mofetil, and prednisolone [48].

### 2.2. Serum Collection and FTIR Spectral Acquisition

Pre-transplant serum samples were collected on the day of kidney transplantation, before graft implantation. For FTIR analysis, 25 μL of serum diluted 1:10 in Milli-Q water (Merck KGaA, Darmstadt, Germany) were deposited onto a 96-well silicon microplate and dehydrated under vacuum in a desiccator for 150 min. Spectral acquisition was performed using a Vertex 70 FTIR spectrometer (Bruker, Ettlingen, Germany) equipped with an HTS-XT accessory (Bruker, Ettlingen, Germany). Each spectrum consisted of 64 co-added scans acquired in transmission mode over the 400–4000 cm^−1^ range at a spectral resolution of 2 cm^−1^. The first well of each 96-well plate was left empty and used for background acquisition according to the manufacturer’s instructions for the HTS-XT system.

For downstream analysis, the spectral regions from 600 to 1900 cm^−1^ and from 2800 to 3400 cm^−1^ were considered, corresponding to the main biochemical fingerprint and high-wavenumber regions of interest.

### 2.3. Spectral Quality Control and Exclusion Criteria

Initial spectral preprocessing was performed in OPUS software (6.5, Bruker, Ettlingen, Germany) and included atmospheric compensation and removal of bands associated with CO_2_ and H_2_O. Further preprocessing was carried out after atmospheric compensation in Orange3 version 3.40.0 (Bioinformatics Laboratory, University of Ljubljana, Ljubljana, Slovenia). The preprocessing strategies evaluated included Rubber Band baseline correction, vector normalization, and first- and second-derivative transformation using a Savitzky–Golay filter, with and without normalization.

Spectral quality control was performed on the full FTIR cohort using the fingerprint region (900–1800 cm^−1^). A total of 80 spectra were initially evaluated. Quality assessment combined complementary metrics capturing global conformity to the cohort spectral profile, local spectral instability, baseline behaviour, signal-to-noise characteristics, and multivariate deviation.

For each spectrum, cosine similarity to the cohort median spectrum was calculated after standard normal variate (SNV) transformation within the fingerprint region. To assess discordance in adjacent-point behaviour, an additional similarity metric was computed from the first-difference profile of the raw spectrum relative to the cohort median first-difference profile. Signal-to-noise ratio (SNR) in the Amide I region was estimated as the peak-to-trough amplitude within 1585–1705 cm^−1^ divided by the standard deviation in the 1800–1900 cm^−1^ interval. Abrupt local irregularities were quantified as the number of points in the first-difference profile with robust z-score > 5. Baseline distortion was summarized by a drift index defined as the absolute difference between the mean SNV intensity in the 1750–1800 cm^−1^ and 900–950 cm^−1^ intervals. Multivariate deviation was further assessed by principal component analysis (PCA), and each spectrum was characterized by its squared distance in the 5-component PCA score space.

The spectral quality-control workflow was implemented in Python 3.13.5 and included similarity-based metrics, signal-to-noise estimation, spike burden assessment, drift quantification, and PCA-based outlier detection. Spectra were not excluded on the basis of a single metric alone. Exclusion was considered only when adverse behavior was concordant across several independent quality-control domains. No single numerical threshold was used in isolation to exclude spectra. The predefined exclusion rule required concordant extreme deviation across independent quality-control domains, including similarity to the cohort median spectrum, first-difference similarity, spike burden, baseline drift, signal-to-noise assessment, and PCA-based distance. The excluded spectrum represented the most extreme observation across several of these metrics simultaneously, rather than an isolated abnormal value in a single quality-control domain.

### 2.4. Exploratory and Machine-Learning Analyses

Exploratory analysis of the spectral data included t-distributed stochastic neighbor embedding (t-SNE), a nonlinear dimensionality-reduction method used to visualize high-dimensional spectral relationships in a low-dimensional space while preserving local neighborhood structure.

For supervised modelling, Naïve Bayes classifiers were used to evaluate whether pre-transplant serum FTIR spectra could discriminate recipients who subsequently developed biopsy-proven rejection from those who did not. Spectral preprocessing was applied to the full FTIR spectrum before extraction of the predefined spectral regions. The evaluated regions were 600–1900 cm^−1^, 2800–3400 cm^−1^, and the combined 600–1900 + 2800–3400 cm^−1^ region. The preprocessing strategies assessed included Rubber Band baseline correction, vector normalization, first derivative transformation, second derivative transformation, and derivative transformation followed by normalization.

The initial FCBF analysis was used as an exploratory feature-selection workflow to identify informative preprocessing and spectral-region configurations. Because feature selection performed before cross-validation can introduce information leakage and may produce optimistic performance estimates, these non-nested FCBF results were reclassified as exploratory and are reported in the Supplementary Material for transparency.

To obtain leakage-aware internal performance estimates, the main supervised analysis in the revised manuscript used a nested feature-selection strategy within Leave-One-Out Cross-Validation (LOOCV). In each LOOCV iteration, one recipient was held out as the test observation, and all feature-selection and model-training steps were performed exclusively on the remaining training observations. Fast Correlation-Based Filter (FCBF) feature selection was therefore repeated independently within each training fold, and the features selected in that fold were then used to train the Naïve Bayes classifier and predict the held-out recipient. The held-out sample was not used during feature selection, model fitting, or parameter estimation.

Model performance was assessed using out-of-fold LOOCV predictions and summarized by the area under the receiver operating characteristic curve (AUC), accuracy, sensitivity, specificity, balanced accuracy, and F1-score. Bootstrap resampling of the out-of-fold predictions was used to estimate 95% confidence intervals for the main performance metrics.

To further assess robustness, permutation testing was performed for the best-performing nested model using 1000 label-randomized repetitions. In each permutation, rejection labels were randomly reassigned while preserving the analytical workflow, including nested FCBF feature selection and LOOCV classification. The empirical *p*-value was calculated as (*r* + 1)/(*n* + 1), where r is the number of permutations with AUC equal to or greater than the observed AUC and *n* is the number of permutations.

An exploratory clinical-only comparison was also performed using pre-transplant clinical and immunological variables available in the dataset, including age, sex, PRA-CDC max, donor-specific antibody status, retransplantation status, donor type, and total HLA mismatch burden. A regularized logistic regression model was evaluated using LOOCV, with missing-value imputation and scaling performed within each training fold to avoid information leakage. A complete-case sensitivity analysis was also performed among recipients with complete clinical covariates. Finally, an exploratory clinical-plus-FTIR model was assessed by combining the nested FTIR out-of-fold probability with the available clinical variables. These analyses were considered exploratory and were used only to contextualize the FTIR signal relative to conventional baseline variables.

**Figure 1:**
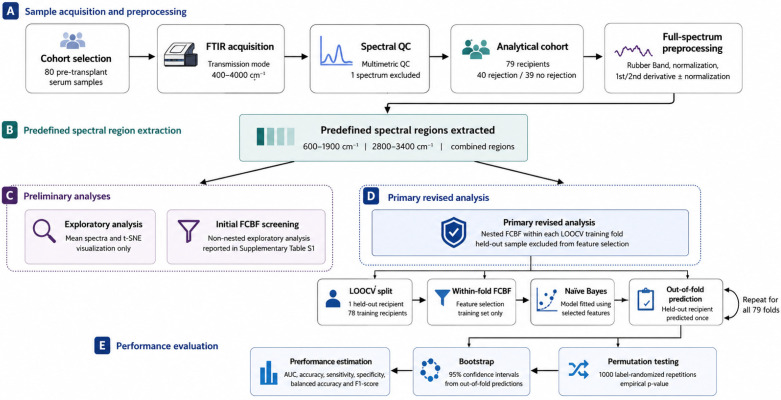
Analytical workflow of the pre-transplant serum FTIR machine-learning pipeline. The workflow summarizes cohort selection, serum collection, FTIR acquisition, spectral quality control, preprocessing of the full spectrum, extraction of predefined spectral regions, exploratory analysis, initial non-nested FCBF screening, nested FCBF-LOOCV classification, Naïve Bayes prediction, permutation testing, and performance reporting. FCBF, Fast Correlation-Based Filter; FTIR, Fourier-transform infrared spectroscopy; LOOCV, leave-one-out cross-validation.

The primary FTIR analytical workflow is summarized in Figure 1. Briefly, pre-transplant serum samples underwent FTIR acquisition, multimetric spectral quality control, full-spectrum preprocessing, predefined spectral-region extraction, exploratory visualization, and supervised classification. The initial non-nested FCBF analysis was retained as an exploratory screening step and is reported in the Supplementary Material for transparency. The primary revised analysis used FCBF feature selection nested within each LOOCV training fold, followed by Naïve Bayes classification of the held-out recipient, out-of-fold performance estimation, bootstrap confidence intervals, and permutation testing. The exploratory clinical-only and clinical-plus-FTIR comparisons were performed separately to contextualize the FTIR signal relative to available baseline clinical variables and were not part of the primary FTIR feature-selection pipeline.

### 2.5. Statistical Analysis

Baseline demographic, clinical, and immunologic variables were analyzed to characterize the study cohort and to assess potential between-group differences. Statistical analyses of clinical and demographic variables were performed using GraphPad Prism version 8.2.0 (GraphPad Software, San Diego, CA, USA). Continuous variables were expressed as median and interquartile range and compared using the Mann–Whitney U test. Binary categorical variables were expressed as number and percentage and compared using Fisher’s exact test. Dialysis modality, as a categorical variable with more than two levels, was compared using the Fisher–Freeman–Halton exact test. A two-sided *p*-value < 0.05 was considered statistically significant.

## 3. Results

### 3.1. Baseline Demographic, Clinical, and Immunological Characteristics of the Cohort

Eighty pre-transplant serum FTIR spectra were initially evaluated. After spectral quality control, one spectrum was excluded, leaving 79 patients for downstream analyses. The baseline demographic, clinical, and immunologic characteristics of this analytical cohort are summarized in Table 1. Because the aim of the study was to evaluate whether pre-transplant serum FTIR profiles could identify patients at risk of post-transplant rejection, only variables available at or before transplantation were considered in the baseline description. These included age, sex, dialysis modality, transplant history, donor type, and pre-transplant immunological risk indicators. This baseline overview was used to contextualize the cohort and to assess whether significant differences between groups were already apparent before spectral analysis. No statistically significant differences were observed between recipients with and without subsequent rejection for the baseline clinical and immunological variables presented in Table 1. The number of HLA-DR mismatches was numerically higher in recipients who developed rejection, but this difference did not reach statistical significance.

In addition to the binary rejection endpoint, rejection phenotype was characterized among recipients with biopsy-proven rejection. Among the 40 recipients who developed biopsy-proven rejection, 22 had humoral rejection, 13 had cellular rejection, and 5 had mixed rejection. Given the limited number of cases within each rejection subtype, subtype-specific machine-learning models were not performed. Among recipients who developed biopsy-proven rejection, the median time from transplantation/pre-transplant serum sampling to rejection was 98.5 days (IQR 24.5–719.0; range 3–2291 days). Twenty rejection episodes occurred within 90 days, 22 within 180 days, and 26 within the first year after transplantation, whereas 14 occurred after one year. When stratified by rejection subtype, median time to rejection was 327.0 days (IQR 23.5–826.0) for humoral rejection, 45.0 days (IQR 35.0–326.0) for cellular rejection, and 107.0 days (IQR 62.0–291.0) for mixed rejection.

### 3.2. Spectral Quality-Control Assessment

Of the 80 spectra initially assessed, only one fulfilled the predefined multimetric spectral quality-control exclusion criterion. Within the predefined quality control (QC) interval (900–1800 cm^−1^), this spectrum showed the most extreme QC profile in the cohort. Quantitatively, it had the lowest cosine similarity to the cohort median SNV spectrum (0.8220), the lowest cosine similarity for the first-difference profile (0.0369), the highest spike count (213), the highest drift index (2.1596), and the largest PCA score distance (PCA D^2^ = 46.5786). Its Amide I signal-to-noise ratio was 27.9932.

**Figure 2:**
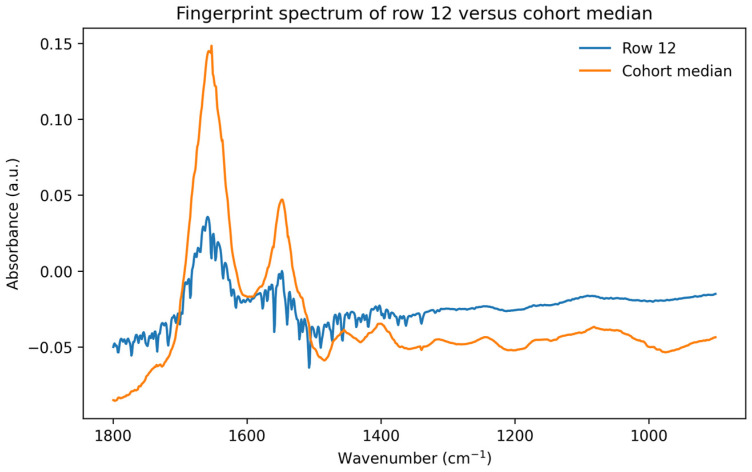
Raw FTIR spectrum of excluded spectra (row 12) overlaid with the cohort median spectrum in the fingerprint region (900–1800 cm^−1^). The excluded spectrum shows marked deviation from the dominant cohort spectral profile.

**Figure 3:**
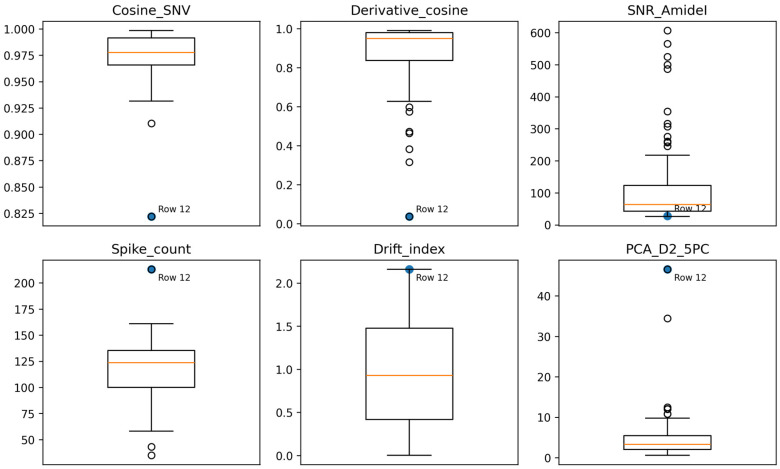
Distributions of SNV-based cosine similarity to the cohort median spectrum, cosine similarity of the first-difference profile, Amide I signal-to-noise ratio, spike count, drift index, and PCA score distance (D^2^, 5 PCs). Excluded spectra (Row 12) consistently occupied an extreme position across multiple independent QC metrics and was therefore excluded from downstream analysis.

Visual inspection of the raw fingerprint spectrum was consistent with these quantitative findings (Figure 2). In addition, when the distributions of the main QC metrics were examined across all spectra, this spectrum consistently occupied an extreme position across multiple independent domains rather than showing a single isolated abnormality (Figure 3).

Taken together, these findings indicate that the excluded spectrum was not identified on the basis of one isolated deviation, but because it represented the single most extreme multimetric QC outlier in the cohort. On this basis, the spectrum was excluded from downstream analyses.

After exclusion, 79 spectra remained for subsequent analyses, including 40 spectra in the rejection group and 39 in the no-rejection group. Cohort-level summary metrics changed modestly after exclusion, with mean SNV cosine similarity increasing from 0.9737 to 0.9757, mean first-difference cosine similarity increasing from 0.8711 to 0.8814, mean spike count decreasing from 118.7500 to 117.5570, mean drift index decreasing from 0.9432 to 0.9278, and the maximum PCA score distance decreasing from 46.5786 to 34.1402. These changes are consistent with removal of the most extreme multimetric QC outlier in the cohort. The retained set of 79 spectra was used for all subsequent exploratory and supervised analyses.

### 3.3. Exploratory Spectral Analysis

Following spectral quality control, exploratory spectral analysis was performed on the 79 retained spectra. Multiple preprocessing strategies and spectral-region configurations were evaluated during exploratory analysis, including the 600–1900 cm^−1^ and 2800–3400 cm^−1^ regions under different combinations of baseline correction and normalization. For representative exploratory presentation, the 600–1900 cm^−1^ region after Rubber Band baseline correction and vector normalization was selected, as this preprocessing provided the most interpretable visualization of group-level spectral structure without overemphasizing global intensity effects.

**Figure 4:**
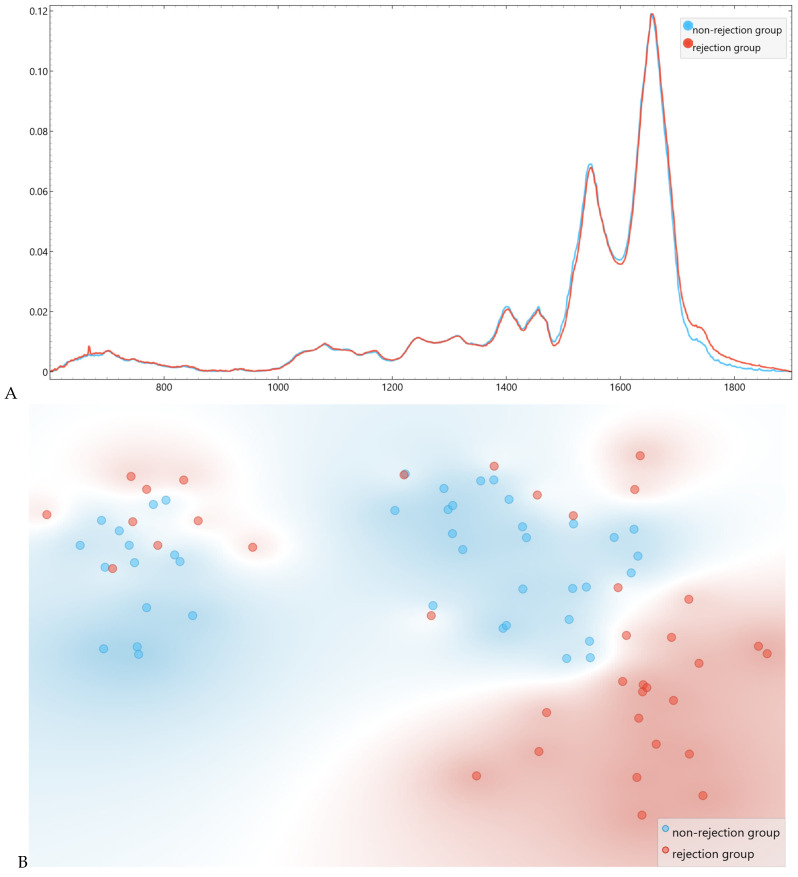
Exploratory spectral analysis of pre-transplant serum FTIR data after quality control. (**A**) Group mean FTIR spectra of patients with and without subsequent biopsy-proven rejection in the 600–1900 cm^−1^ region after Rubber Band baseline correction and vector normalization. The spectra show broad overall overlap, with localized differences distributed across the fingerprint region. (**B**) Two-dimensional t-distributed stochastic neighbor embedding (t-SNE) projection of the same preprocessed spectra. Partial local grouping according to rejection status was observed, although substantial overlap between classes remained. Background shading represents a two-dimensional kernel density estimate of class distribution in the t-SNE embedding and is provided only as a visual aid; it was not used for model training, feature selection, or performance estimation.

Comparison of the group mean spectra under this preprocessing condition showed broad overall overlap between patients with and without subsequent biopsy-proven rejection, indicating that no single visually dominant spectral feature separated the two groups (Figure 4A). However, localized differences were observed across several portions of the fingerprint region, suggesting that class-related information was distributed across the spectral profile rather than concentrated in an isolated band. This pattern is consistent with a subtle multivariate signal rather than a single major spectral shift.

To further explore the global organization of the dataset, t-SNE was applied to the same preprocessed spectra. The resulting two-dimensional projection suggested partial local grouping according to rejection status, with areas relatively enriched for one class or the other, although substantial overlap between groups remained (Figure 4B). Thus, the exploratory analysis supported the presence of class-related spectral structure, while also indicating that unsupervised visualization alone was insufficient for complete separation of the two groups.

Taken together, these exploratory findings suggest that the discriminatory information contained in pre-transplant serum FTIR spectra was subtle, distributed across multiple variables, and not fully resolved by unsupervised visualization alone. This pattern supported progression to supervised machine-learning analysis.

### 3.4. Machine-Learning Performance, Feature Selection, and Model Robustness

Following exploratory analysis, supervised classification was performed to assess whether pre-transplant serum FTIR spectra could discriminate patients who subsequently developed biopsy-proven rejection from those who did not. Multiple preprocessing strategies, spectral-region configurations, and feature-selection conditions were evaluated using Naïve Bayes models with LOOCV. The results are presented below according to exploratory preprocessing-dependent performance, initial non-nested FCBF screening, nested FCBF-LOOCV classification, permutation-based robustness testing, and stability of the selected spectral variables.

#### 3.4.1. Model Performance Across Preprocessing Pipelines

Naïve Bayes models were first compared across preprocessing pipelines and spectral-region configurations without feature selection. Classification performance varied substantially according to both the spectral region analyzed and the preprocessing strategy applied (Table 2), indicating that preprocessing had a marked influence on model behavior.

Within the 600–1900 cm^−1^ region, baseline correction or normalization alone yielded relatively modest discrimination, with AUC values of 0.706 and 0.699, respectively. Combining Rubber Band baseline correction with vector normalization improved performance (AUC = 0.756; accuracy = 0.734; sensitivity = 0.734; specificity = 0.736). Derivative-based preprocessing also influenced classification results. First derivative alone yielded an AUC of 0.728, whereas first derivative combined with normalization yielded an AUC of 0.714. Second derivative alone achieved an AUC of 0.749, but the combination of second derivative and normalization provided the strongest performance in this region, with an AUC of 0.797, an accuracy of 0.785, a sensitivity of 0.785, and a specificity of 0.787.

A similar pattern was observed in the 2800–3400 cm^−1^ region. Baseline correction alone yielded an AUC of 0.728, while vector normalization alone performed less well (AUC = 0.674). Rubber Band baseline correction combined with vector normalization yielded an AUC of 0.711. First derivative alone achieved an AUC of 0.744 and an accuracy of 0.772, whereas first derivative combined with normalization reduced performance (AUC = 0.652). Second derivative alone yielded an AUC of 0.776, but second derivative combined with normalization provided the strongest performance in this region, achieving the highest AUC observed in Table 2 (AUC = 0.840), together with an accuracy of 0.823, a sensitivity of 0.823, and a specificity of 0.825.

When the two spectral regions were combined (600–1900 + 2800–3400 cm^−1^), the same overall trend persisted. Baseline correction alone yielded an AUC of 0.722, while vector normalization alone yielded an AUC of 0.689. Rubber Band baseline correction combined with vector normalization improved discrimination (AUC = 0.781; accuracy = 0.734; sensitivity = 0.734; specificity = 0.736). First derivative and first derivative combined with normalization yielded AUC values of 0.742 and 0.746, respectively. Second derivative alone yielded an AUC of 0.760, whereas second derivative combined with normalization again produced the strongest performance in the combined-region analysis, with an AUC of 0.815, an accuracy of 0.797, a sensitivity of 0.797, and a specificity of 0.800.

Taken together, these results show that preprocessing choice and spectral-region selection materially influenced classifier behaviour. In particular, derivative-based normalized spectra were among the strongest preprocessing strategies in the exploratory comparison. Because multiple preprocessing pipelines were evaluated, these results were interpreted as an exploratory preprocessing comparison and were followed by feature-selection analyses. The initial non-nested FCBF analysis is reported in the Supplementary Material for transparency, whereas the primary revised performance estimates are based on nested FCBF-LOOCV.

#### 3.4.2. Initial Exploratory Non-Nested FCBF Analysis

Application of FCBF feature selection in the initial workflow suggested that dimensionality reduction could improve Naïve Bayes classifier performance across several preprocessing pipelines and spectral-region configurations. In this exploratory analysis, the highest AUC values were observed after FCBF selection, particularly in derivative-normalized and combined-region models. These findings were useful for identifying promising preprocessing strategies and spectral regions for further evaluation.

However, because this initial FCBF analysis was performed as a non-nested exploratory workflow, feature selection may have used information from the full dataset before LOOCV performance estimation. This design can introduce information leakage and may lead to optimistic estimates of model performance. For this reason, the original non-nested FCBF results are no longer used as the primary estimates of model generalization performance. They are reported in Supplementary Table S1 for transparency, while the revised primary analysis was performed using FCBF nested within each LOOCV training fold, as described below.

#### 3.4.3. Nested FCBF-LOOCV Model Performance

Because feature selection performed before cross-validation can lead to optimistic performance estimates, the main supervised analysis was repeated using FCBF feature selection nested within each LOOCV training fold. Under this corrected validation framework, the held-out observation was not used during feature selection, model fitting, or parameter estimation.

The best-performing nested model was obtained using second derivative transformation followed by normalization in the combined 600–1900 + 2800–3400 cm^−1^ region. This model achieved an AUC of 0.837, with an accuracy of 0.747, sensitivity of 0.675, specificity of 0.821, balanced accuracy of 0.748, and F1-score of 0.730. The corresponding confusion matrix included 27 true-positive, 13 false-negative, 32 true-negative, and 7 false-positive classifications. The highest-ranked nested models are summarized in Table 3.

Across the evaluated pipelines, derivative-based preprocessing remained among the strongest approaches, although the corrected nested estimates were lower than those obtained in the initial non-nested feature-selection analysis. This reduction is methodologically expected, because feature selection was repeated independently within each training fold and the held-out observation remained fully independent from the feature-selection step. The results therefore support the presence of a discriminatory pre-transplant FTIR signal while providing a more conservative estimate of internal model performance.

#### 3.4.4. Permutation-Based Robustness Analysis

Permutation testing was performed to assess whether the performance of the best nested FCBF-LOOCV model exceeded what would be expected by chance in a high-dimensional dataset. The best nested model, based on second derivative transformation followed by normalization in the combined 600–1900 + 2800–3400 cm^−1^ region, was therefore evaluated using 1000 label-randomized repetitions while preserving the same analytical workflow.

The observed AUC of 0.837 was higher than all AUC values obtained after label randomization. Across the permuted models, the mean AUC was 0.470 and the median AUC was 0.468. The central 95% of the null AUC distribution ranged from 0.265 to 0.661, and the maximum permuted AUC was 0.803. No permutation achieved an AUC equal to or greater than the observed value. To avoid reporting a zero probability from a finite number of permutations, the empirical *p*-value was calculated using the correction (*r* + 1)/(*n* + 1), where r is the number of permutations with AUC greater than or equal to the observed AUC and *n* is the total number of permutations. With r = 0 and *n* = 1000, this corresponded to an empirical *p*-value of 0.000999. Bootstrap analysis of the observed out-of-fold predictions yielded a 95% confidence interval for the observed AUC of 0.741–0.918. The robustness results for the best nested model are summarized in Table 4.

Together, these findings indicate that the corrected nested model performed substantially above chance expectation, while also showing the uncertainty expected from a modest single-centre cohort.

#### 3.4.5. Exploratory Clinical-Only and Clinical-Plus-FTIR Comparison

To assess whether the FTIR-based model provided information beyond available pre-transplant clinical and immunological variables, an exploratory clinical-only model was evaluated using age, sex, PRA-CDC max, donor-specific antibody status, retransplantation status, donor type, and total HLA mismatch burden. The clinical-only model showed limited discrimination, with an AUC of 0.563, accuracy of 0.608, sensitivity of 0.550, and specificity of 0.667. A complete-case sensitivity analysis restricted to recipients with complete clinical covariates yielded a similar AUC of 0.575.

For comparison, the best nested FTIR-only model achieved an AUC of 0.837. An exploratory clinical-plus-FTIR model, incorporating the nested FTIR out-of-fold probability together with the available clinical variables, achieved an AUC of 0.739 and did not improve discrimination over the FTIR-only model. These findings suggest that, within the constraints of the available clinical variables and the modest cohort size, the FTIR signal was not simply recapitulating the available conventional clinical and immunological markers. However, formal assessment of incremental value will require larger cohorts with more complete clinical covariate capture.

#### 3.4.6. Stability and Spectral Distribution of Selected Features

The variables retained by nested FCBF in the best-performing model were distributed across both the fingerprint and high-wavenumber regions. Because feature selection was repeated independently within each LOOCV training fold, feature stability was assessed by calculating the frequency with which each wavenumber was selected across the 79 training folds. The initial non-nested FCBF feature list from the exploratory analysis is provided separately in Supplementary Table S2, whereas the main manuscript focuses on fold-wise feature stability from the leakage-aware nested analysis.

The most frequently selected variables included wavenumbers around ≈630 cm^−1^ and ≈676 cm^−1^ in the lower fingerprint region, ≈1141 cm^−1^ and ≈1284 cm^−1^ in the fingerprint region, and ≈2832 cm^−1^, ≈3042 cm^−1^, and ≈3329 cm^−1^ in the high-wavenumber region. This distribution indicates that the discriminatory signal was not confined to a single isolated spectral band but instead reflected a restricted multivariate pattern across complementary spectral domains. The most frequently selected wavenumbers in the FCBF-LOOCV model are presented in Table 5.

Wavenumbers are reported from the best-performing nested model using second derivative plus normalization in the combined 600–1900 and 2800–3400 cm^−1^ regions. Selection frequency indicates the proportion of LOOCV training folds in which each variable was selected by FCBF.

These assignments should be interpreted cautiously, particularly because second-derivative FTIR variables reflect local spectral curvature and because serum FTIR bands represent overlapping contributions from proteins, lipids, carbohydrates, glycoproteins, and other circulating biochemical components. Therefore, the selected wavenumbers should be considered hypothesis-generating spectral markers rather than direct identification of specific molecules or mechanisms.

## 4. Discussion

The revised analysis indicates that pre-transplant serum FTIR spectra contain an internally validated spectral signal associated with subsequent biopsy-proven rejection. This is the principal finding of the work. Importantly, after nesting FCBF feature selection within each LOOCV training fold, model performance remained above chance but was more conservative than in the initial non-nested analysis. The best corrected model achieved an AUC of 0.837, with specificity of 0.821 and sensitivity of 0.675, supporting the presence of a relevant but not definitive discriminatory signal.

This observation should not be interpreted as evidence that rejection can be diagnosed before transplantation. A more biologically coherent interpretation is that pre-transplant serum FTIR may capture a baseline spectral biochemical signature associated with susceptibility to subsequent rejection after graft implantation. Clinically, such a signal should not be interpreted as a reason to withhold transplantation, but rather as a possible means of identifying recipients who may benefit from closer post-transplant surveillance and more individualized follow-up if the finding is externally validated. Such a view is more consistent with current understanding of kidney transplantation, in which rejection risk emerges from the interaction of recipient immunologic status, donor–recipient compatibility, graft-related factors, and post-transplant immune activation rather than from any single pre-existing determinant [1,2,4,5,13].

This distinction is clinically important. Contemporary rejection assessment remains fundamentally anchored to biopsy-based interpretation within the Banff framework, even as Banff continues to evolve through reappraisal of microvascular inflammation, molecular pathology, and transcript-based biopsy diagnostics. At the same time, kidney biopsy is invasive, constrained by sampling limitations and interpretive variability, and associated with a measurable complication burden. For these reasons, the search for non-invasive tools that can complement histology remains a major priority in transplantation medicine. Yet, despite substantial progress, currently available biomarker strategies have not fully resolved the need for robust, scalable, and clinically deployable tools across the transplant timeline. Even the most intensively studied approaches, including donor-derived cell-free DNA, blood transcriptomic assays, urinary chemokines, extracellular vesicles, and targeted molecular panels, continue to face limitations related to context of use, reproducibility, standardization, and incremental value over established clinical and immunologic monitoring. Within this landscape, FTIR should be positioned not as a substitute for biopsy or conventional immunologic assessment, but as a potentially complementary approach for integrated biochemical risk phenotyping [1,2,5,49,50,51,52,53].

One of the clearest methodological messages from the present study is that classification performance depended strongly on spectral preprocessing. Across the region-specific analyses, derivative-based preprocessing combined with normalization consistently outperformed baseline correction, normalization on their own, and derivative preprocessing without normalization. This was particularly evident for second derivative + normalization, which yielded the strongest region-specific performance both before and after feature selection. In FTIR-based biomarker work, preprocessing is sometimes treated as a technical preparatory step, but the present data indicate that it is instead a major determinant of model behavior. In practical terms, this means that preprocessing acted as a major determinant of classifier behaviour and therefore required transparent reporting and leakage-aware validation. For this reason, the revised manuscript distinguishes between exploratory preprocessing comparisons, the initial non-nested FCBF screening reported in the Supplementary Material, and the primary nested FCBF-LOOCV analysis reported in the main Results. That conclusion is highly consistent with broader biospectroscopy literature emphasizing the central importance of preprocessing optimization, derivative treatment, normalization strategy, and transparent analytical reporting in biofluid-based machine-learning studies [31,34,36,54,55,56,57].

The region-specific results are also informative. In the absence of feature selection, the strongest region-specific performance was observed in the 2800–3400 cm^−1^ domain using second derivative plus normalization, which achieved the highest AUC among the region-specific non-feature-selected models. In the 600–1900 cm^−1^ region, the same preprocessing strategy also produced the strongest performance within that domain. This is noteworthy because FTIR studies of biological fluids often privilege the fingerprint region as the principal source of discriminatory information. By contrast, the present findings indicate that the high-wavenumber region also carried substantial predictive signal. This is biologically relevant because the two spectral windows reflect partly distinct biochemical domains: the fingerprint region contains complex contributions from phosphate-containing compounds, carbohydrates, C–O stretching modes, and protein-related amide/carbonyl bands, whereas the high-wavenumber region is more strongly influenced by aliphatic C–H stretching vibrations and, in its upper range, by N–H/O–H stretching contributions. Taken together, these findings suggest that rejection-associated biochemical susceptibility is reflected across more than one spectral compartment and that the underlying signal is distributed and multivariate rather than confined to one dominant spectral feature [31,32,35,37,38,41,47].

A central methodological finding of the revised analysis is that model performance remained meaningful after correction for feature-selection leakage, although the estimates were more conservative than in the initial non-nested analysis. When FCBF was nested within each LOOCV training fold, the best model achieved an AUC of 0.837 rather than the higher values observed when feature selection was applied before cross-validation. This reduction should not be viewed as a weakness of the revised analysis. Rather, it provides a more realistic estimate of internal performance by ensuring that the held-out observation did not influence the selected feature set.

The initial non-nested FCBF analysis remains informative as an exploratory screening of preprocessing strategies and spectral regions and is therefore provided in the Supplementary Material for transparency. However, the revised nested FCBF-LOOCV results are used as the primary estimates of model performance and form the basis of the revised interpretation.

The permutation analysis further supports the presence of a non-random signal. Across 1000 label-randomized repetitions, no permuted model reached the observed AUC, resulting in an empirical *p*-value of approximately 0.001. This finding argues against the interpretation that the observed discrimination is solely an artefact of high dimensionality, small sample size, or the analytical pipeline. Nevertheless, the bootstrap confidence interval around the AUC remained relatively wide, reflecting the limited size of the cohort and reinforcing the need for external validation [31,32,37,38,58,59,60].

The selected spectral variables should be interpreted cautiously. The most frequently selected wavenumbers were distributed across both the fingerprint and high-wavenumber regions, suggesting that the classification signal is multivariate rather than attributable to a single isolated biochemical band. However, FTIR spectra of serum represent overlapping contributions from proteins, lipids, carbohydrates, glycoproteins, and other circulating molecular components. Therefore, the present results do not identify specific molecular mechanisms of rejection susceptibility. Instead, they support the hypothesis that pre-transplant serum contains a spectral biochemical signature associated with later biopsy-proven rejection. Orthogonal validation using proteomics, metabolomics, lipidomics, or immune profiling will be required to define the biological basis of this signal.

The exploratory analyses support this view rather than contradict it. Group mean spectra showed broad overlap, and t-SNE revealed only partial local grouping with persistent overlap between classes. These results indicate that the pre-transplant serum signature associated with later rejection was subtle and not fully separable by unsupervised visualization alone. The continuity between the exploratory and supervised analyses is therefore an important strength of the study: the signal was present but weakly organized in unsupervised space and more efficiently resolved after preprocessing optimization and supervised dimensionality reduction [31,32,37,38,58,59,60,61].

From a nephrology and transplant perspective, the timing of sampling is one of the most distinctive aspects of this study. Most currently research non-invasive biomarkers in kidney transplantation are framed around post-transplant surveillance, when the graft is already in place and injury-related signals may already be evolving. By contrast, the present study evaluated serum collected on the day of transplantation, before graft implantation. This pre-transplant window is clinically attractive because it addresses a time point at which conventional injury markers are not yet informative for the future recipient. In that sense, the present work is not proposing FTIR as a substitute for diagnostic biopsy, nor as a replacement for post-transplant monitoring biomarkers. Rather, it suggests a different niche: baseline biochemical risk phenotyping before transplantation. Thus, the present study extends previous post-transplant FTIR work by moving the analytical question to baseline pre-transplant risk phenotyping [5,6,62].

The completed baseline comparison helps contextualize this interpretation. In the variables assessed at or before transplantation, no statistically significant between-group differences were identified for age, sex, dialysis modality, retransplantation status, donor type, PRA-CDC max, pre-transplant donor-specific antibodies, pre-transplant anti-HLA antibodies, or total HLA mismatch burden. This does not prove biological equivalence between groups, nor does it demonstrate that the FTIR signal is independent of classical immunologic risk. However, it does reduce the likelihood that the spectroscopic findings simply reflect a gross imbalance in one of the available baseline variables. In a proof-of-concept study of this size, that is an important point. The present findings are therefore more consistent with integrated biochemical patterning than with a trivial single-variable surrogate. Even so, this issue should be interpreted cautiously until larger studies can formally test incremental value over established pre-transplant risk markers [7,9,11,63,64,65,66].

The exploratory clinical-only comparison provides an additional context for interpreting the FTIR signal. Using the available pre-transplant clinical and immunological variables, the clinical-only model showed limited discrimination, whereas the best nested FTIR-only model achieved substantially higher internal performance. Importantly, the exploratory clinical-plus-FTIR model did not improve over the FTIR-only model. This finding should not be interpreted as definitive evidence that FTIR has independent incremental clinical value, because the cohort was modest, several clinical covariates were incomplete, and the combined model was exploratory. However, it suggests that the FTIR signal was not simply reproducing the discriminatory information contained in the available conventional baseline variables. Larger cohorts with complete clinical covariate capture will be required to formally test incremental value using predefined clinical-only, FTIR-only, and combined models.

The study results also extend our group’s previous work in a meaningful way. Earlier studies showed that FTIR signatures combined with machine learning could discriminate kidney allograft rejection in post-transplant settings, that serum molecular fingerprinting was associated with cellular rejection, and that transplant-related biofluids other than serum could also yield clinically relevant spectroscopic patterns. The current study differs in a conceptually important respect: it moves the analytical focus to the pre-transplant period. This is not a small technical modification. It changes the biological question from contemporaneous rejection-associated profiling to baseline risk phenotyping before graft implantation. If confirmed, such an approach could complement conventional pre-transplant assessment by providing a rapid, label-free, high-throughput, and systems-level biochemical readout that may capture aspects of susceptibility not fully resolved by standard clinical and immunologic variables alone. In practical terms, its most plausible role would be to support closer post-transplant surveillance in recipients identified as being at higher risk, rather than to alter transplant eligibility on the basis of spectroscopy alone [45,46,47].

Several limitations should be emphasized. First, this was a retrospective single-centre proof-of-concept study with a modest analytical cohort. Although the revised nested FCBF-LOOCV analysis and permutation testing reduce the risk of overoptimistic interpretation, they do not replace external validation. Second, the primary endpoint combined antibody-mediated, T cell-mediated, and mixed rejection phenotypes. In the present cohort, rejection subtypes included 22 humoral, 13 cellular, and 5 mixed rejection cases. These numbers were sufficient for descriptive reporting but insufficient for reliable subtype-specific machine-learning modelling. Third, although time to rejection was reported descriptively, the present study did not model rejection as a time-to-event outcome and did not account for censoring in the no-rejection group. The results should therefore be interpreted as an association with biopsy-proven rejection during follow-up rather than as prediction of rejection within a predefined temporal window. Future studies should evaluate pre-transplant FTIR signatures using survival-analysis frameworks. Fourth, the study did not include independent metabolomic, proteomic, lipidomic, or immunological validation of the selected FTIR features. As a result, the biochemical interpretation remains hypothesis-generating. Fifth, although baseline clinical and immunological variables were described, the study was not powered to definitively establish the incremental value of FTIR over conventional pre-transplant risk markers. This question should be addressed in larger cohorts using predefined clinical-only, FTIR-only, and combined clinical-plus-FTIR models [67,68]. Because serum FTIR profiling was performed before graft implantation, the primary aim of this study was to evaluate a pre-transplant spectral risk signal rather than the effect of post-transplant immunosuppression. Although immunosuppression followed institutional standard-of-care practice, the present analysis was not designed or powered to assess whether specific induction agents, maintenance regimens, drug exposure, or subsequent treatment modifications influenced the association between the pre-transplant FTIR signature and later biopsy-proven rejection. Cold ischemia time was not available in a sufficiently complete and standardized format for inclusion in the baseline comparative table or exploratory clinical-only modelling.

Despite these limitations, the revised analysis provides a meaningful proof-of-concept. It suggests that pre-transplant serum FTIR contains analyzable information associated with later biopsy-proven rejection, that this information remains detectable after leakage-aware nested validation, and that it can be condensed into a restricted subset of repeatedly selected spectral variables. Future work should validate these findings in larger and independent cohorts, determine whether similar preprocessing and feature-selection strategies remain stable across centres and batches, assess incremental value relative to conventional pre-transplant immunologic assessment, and examine whether distinct rejection phenotypes can be resolved spectroscopically. If these findings are confirmed, pre-transplant serum FTIR could contribute to a broader multimodal framework for rejection-focused precision medicine in kidney transplantation, not as a replacement for biopsy or conventional immunologic testing, but as an additional layer of biologically integrated pre-transplant risk information [37,45].

## 5. Conclusions

This proof-of-concept study shows that pre-transplant serum FTIR spectra contain an internally validated spectral signal associated with subsequent biopsy-proven rejection. After nesting FCBF feature selection within each LOOCV training fold, the best-performing model achieved an AUC of 0.837, and permutation testing supported performance above chance expectation. These findings suggest that FTIR may have value as a complementary, hypothesis-generating approach for pre-transplant biochemical risk phenotyping.

The results should not be interpreted as supporting clinical implementation at this stage. External multicenter validation, locked preprocessing and modelling pipelines, assessment of incremental value over conventional immunological risk markers, and biological validation of the selected spectral features will be required before this approach can be considered for clinical use.

## Figures and Tables

**Table 1:** Baseline demographic, clinical, and immunologic characteristics of the study cohort.

Variable	Overall Cohort (*n* = 79)	No Rejection (*n* = 39)	Rejection (*n* = 40)	*p*-Value
Age at transplantation, years	44.0 (35.5–51.5)	46.0 (38.0–59.0)	42.5 (34.8–49.0)	0.093
Male sex, *n* (%)	49 (62.0)	25 (64.1)	24 (60.0)	0.818
Dialysis modality, *n* (%)				0.759
Hemodialysis	64 (81.0)	31 (79.5)	33 (82.5)	
Peritoneal dialysis	10 (12.7)	6 (15.4)	4 (10.0)	
Preemptive transplantation	5 (6.3)	2 (5.1)	3 (7.5)	
Retransplantation, *n* (%)	11 (13.9)	4 (10.3)	7 (17.5)	0.518
Deceased donor, *n* (%)	72 (91.1)	36 (92.3)	36 (90.0)	1.000
PRA-CDC max, %	4.0 (0.0–43.0)	6.0 (0.0–49.0)	0.0 (0.0–28.0)	0.421
Pre-transplant donor-specific antibodies, *n* (%)	14 (17.7)	5 (12.8)	9 (22.5)	0.378
Pre-transplant anti-HLA antibodies, *n* (%)	54 (68.4)	28 (71.8)	26 (65.0)	0.630
Total number of HLA mismatches (A-B-DR)	4.0 (3.0–5.0)	4.0 (3.0–5.0)	4.0 (3.0–5.0)	0.613
HLA-A mismatches	1.0 (1.0–2.0)	1.0 (1.0–2.0)	1.0 (1.0–2.0)	0.570
HLA-B mismatches	2.0 (1.0–2.0)	2.0 (1.0–2.0)	2.0 (1.0–2.0)	0.921
HLA-DR mismatches	1.0 (1.0–2.0)	1.0 (1.0–2.0)	1.0 (1.0–2.0)	0.096

HLA, human leukocyte antigen; PRA, panel-reactive antibody; CDC, complement-dependent cytotoxicity.

**Table 2:** Exploratory performance of Naïve Bayes models across preprocessing pipelines and spectral-region configurations without FCBF feature selection.

Spectral Region	Preprocessing Pipeline	AUC	Accuracy	Sensitivity	Specificity
600–1900 cm^−1^	Rubber Band baseline correction	0.706	0.696	0.696	0.699
600–1900 cm^−1^	Vector normalization	0.699	0.684	0.684	0.685
600–1900 cm^−1^	Rubber Band + vector normalization	0.756	0.734	0.734	0.736
600–1900 cm^−1^	First derivative	0.728	0.684	0.684	0.686
600–1900 cm^−1^	First derivative + normalization	0.714	0.709	0.709	0.710
600–1900 cm^−1^	Second derivative	0.749	0.709	0.709	0.710
600–1900 cm^−1^	Second derivative + normalization	0.797	0.785	0.785	0.787
2800–3400 cm^−1^	Rubber Band baseline correction	0.728	0.759	0.759	0.764
2800–3400 cm^−1^	Vector normalization	0.674	0.658	0.658	0.660
2800–3400 cm^−1^	Rubber Band + vector normalization	0.711	0.671	0.671	0.675
2800–3400 cm^−1^	First derivative	0.744	0.772	0.772	0.777
2800–3400 cm^−1^	First derivative + normalization	0.652	0.696	0.696	0.699
2800–3400 cm^−1^	Second derivative	0.776	0.734	0.734	0.737
2800–3400 cm^−1^	Second derivative + normalization	0.840	0.823	0.823	0.825
600–1900 + 2800–3400 cm^−1^	Rubber Band baseline correction	0.722	0.709	0.709	0.712
600–1900 + 2800–3400 cm^−1^	Vector normalization	0.689	0.671	0.671	0.674
600–1900 + 2800–3400 cm^−1^	Rubber Band + vector normalization	0.781	0.734	0.734	0.736
600–1900 + 2800–3400 cm^−1^	First derivative	0.742	0.722	0.722	0.724
600–1900 + 2800–3400 cm^−1^	First derivative + normalization	0.746	0.722	0.722	0.723
600–1900 + 2800–3400 cm^−1^	Second derivative	0.760	0.684	0.684	0.686
600–1900 + 2800–3400 cm^−1^	Second derivative + normalization	0.815	0.797	0.797	0.800

**Table 3:** Nested FCBF-LOOCV performance of Naïve Bayes models across selected preprocessing pipelines and spectral regions.

Spectral Region	Preprocessing Pipeline	AUC	Accuracy	Sensitivity	Specificity	Median Selected Features
600–1900 + 2800–3400 cm^−1^	Second derivative + normalization	0.837	0.747	0.675	0.821	7
600–1900 cm^−1^	First derivative	0.822	0.696	0.750	0.641	3
600–1900 + 2800–3400 cm^−1^	First derivative	0.818	0.696	0.750	0.641	3
600–1900 + 2800–3400 cm^−1^	Rubber Band + vector normalization	0.801	0.696	0.625	0.769	1
600–1900 cm^−1^	Second derivative + normalization	0.796	0.747	0.650	0.846	5
2800–3400 cm^−1^	Second derivative + normalization	0.782	0.684	0.575	0.795	4

AUC, area under the receiver operating characteristic curve; FCBF feature selection was performed independently within each training fold. The held-out sample was not used for feature selection, model training, or parameter estimation.

**Table 4:** Robustness assessment of the best nested FCBF-LOOCV model.

Analysis	Result
Best model	Combined 600–1900 + 2800–3400 cm^−1^
Preprocessing	Second derivative + normalization
Classifier	Naïve Bayes
Feature selection	FCBF nested within LOOCV
Observed AUC	0.837
Bootstrap 95% CI for observed AUC	0.741–0.918
Accuracy	0.747
Sensitivity	0.675
Specificity	0.821
Balanced accuracy	0.748
F1-score	0.730
Mean permuted AUC	0.470
Median permuted AUC	0.468
Central 95% interval of permuted AUC distribution	0.265–0.661
Highest permuted AUC	0.803
Permutations with AUC ≥ observed AUC	0/1000
Empirical *p*-value	0.000999

AUC, area under the receiver operating characteristic curve; FCBF, Fast Correlation-Based Filter; LOOCV, Leave-One-Out Cross-Validation. The empirical *p*-value was calculated as (*r* + 1)/(*n* + 1), where r is the number of permutations with AUC greater than or equal to the observed AUC and *n* is the number of permutations.

**Table 5:** Most frequently selected wavenumbers in the best nested FCBF-LOOCV model.

Approximate Wavenumber, cm^−1^	Selection Frequency Across LOOCV Folds
630	98.7%
2832	97.5%
3329	77.2%
3042	72.2%
676	65.8%
1284	60.8%
1141	58.2%

## Data Availability

The data presented in this study are available on request from the corresponding author due to privacy and ethical restrictions related to patient-level clinical and laboratory data.

## Supplementary Materials

The following supporting information can be downloaded at: https://www.mdpi.com/article/10.3390/medsci14030353/s1. Table S1: Exploratory non-nested FCBF feature-selection analysis of Naïve Bayes classifier performance across preprocessing pipelines and spectral-region configurations; Table S2: Spectral distribution of variables selected in the initial exploratory non-nested FCBF analysis. This table reports variables selected in the initial exploratory non-nested FCBF workflow. Because the primary revised model used feature selection nested within each LOOCV fold, the main manuscript reports feature stability as selection frequency across LOOCV folds.

## Abbreviations

The following abbreviations are used in this manuscript:

FTIR	Fourier-transform infrared
DSA	donor-specific anti-HLA antibodies
HLA	Human leukocyte antigen
SNV	Standard normal variate
SNR	Signal-to-noise ratio
PCA	Principal component analysis
t-SNE	t-distributed stochastic neighbor embedding
LOOCV	Leave-One-Out Cross-Validation
FCBF	Fast Correlation-Based Filter
AUC	area under the receiver operating characteristic curve
QC	Quality control
