# Supplementary material for: Risk Phenotyping Before Graft Implantation: FTIR Spectroscopy and Machine Learning for Complementary Risk Stratification in Kidney Transplantation
Source: Med Sci (Basel). 2026 Jun 27;14(3):353. doi: 10.3390/medsci14030353 (PMC13413492; doi:10.3390/medsci14030353)
Supplement: Supplementary file 1 [file medsci-14-00353-s001.zip › medsci-4351722-supplementary.pdf]

Supplementary Materials: Risk Phenotyping Before Graft Implantation: FTIR Spectroscopy and Machine Learning for Complementary Risk Stratification in Kidney Transplantation

Luis Ramalhete, Rúben Araújo, Emanuel Vigia, Miguel Bigotte Vieira, Anibal Ferreira and Cecilia R. C. Calado

Table S1. Exploratory non-nested FCBF feature-selection analysis of Naïve Bayes classifier performance across preprocessing pipelines and spectral-region configurations.

Spectral region	Preprocessing pipeline	FCBF	No. selected features	AUC	Accuracy	Sensitivity	Specificity
600–1900 cm⁻¹	Rubber Band baseline correction	Yes	4	0.878	0.785	0.785	0.785
600–1900 cm⁻¹	Vector normalization	Yes	1	0.710	0.608	0.608	0.611
600–1900 cm⁻¹	Rubber Band + vector normalization	Yes	5	0.894	0.810	0.810	0.812
600–1900 cm⁻¹	First derivative	Yes	3	0.910	0.861	0.861	0.862
600–1900 cm⁻¹	First derivative + normalization	Yes	5	0.908	0.823	0.823	0.825
600–1900 cm⁻¹	Second derivative	Yes	5	0.901	0.835	0.835	0.838
600–1900 cm⁻¹	Second derivative + normalization	Yes	9	0.947	0.886	0.886	0.889
2800–3400 cm⁻¹	Rubber Band baseline correction	Yes	5	0.908	0.861	0.861	0.861
2800–3400 cm⁻¹	Vector normalization	Yes	1	0.644	0.747	0.747	0.753
2800–3400 cm⁻¹	Rubber Band + vector normalization	Yes	6	0.886	0.873	0.873	0.875
2800–3400 cm⁻¹	First derivative	Yes	1	0.739	0.620	0.620	0.623
2800–3400 cm⁻¹	First derivative + normalization	Yes	2	0.627	0.772	0.772	0.775
2800–3400 cm⁻¹	Second derivative	Yes	5	0.909	0.810	0.810	0.813
2800–3400 cm⁻¹	Second derivative + normalization	Yes	7	0.956	0.861	0.861	0.864
600–1900 + 2800–3400 cm⁻¹	Rubber Band baseline correction	Yes	7	0.944	0.911	0.911	0.912

600–1900 + 2800–3400 cm⁻¹	Vector normalization	Yes	1	0.726	0.734	0.734	0.741
600–1900 + 2800–3400 cm⁻¹	Rubber Band + vector normalization	Yes	8	0.960	0.848	0.848	0.849
600–1900 + 2800–3400 cm⁻¹	First derivative	Yes	3	0.910	0.861	0.861	0.862
600–1900 + 2800–3400 cm⁻¹	First derivative + normalization	Yes	5	0.919	0.835	0.835	0.837
600–1900 + 2800–3400 cm⁻¹	Second derivative	Yes	7	0.922	0.873	0.873	0.875
600–1900 + 2800–3400 cm⁻¹	Second derivative + normalization	Yes	10	0.942	0.911	0.911	0.913

This table reports the initial exploratory FCBF analysis in which feature selection was applied before LOOCV. Because non-nested feature selection can introduce information leakage and may yield optimistic performance estimates, these results are reported for transparency and are not used as the primary estimates of model generalization performance. The primary revised analysis is the nested FCBF-LOOCV analysis presented in Table 3.

Table S2. Spectral distribution of variables selected in the initial exploratory non-nested FCBF analysis. This table reports variables selected in the initial exploratory non-nested FCBF workflow. Because the primary revised model used feature selection nested within each LOOCV fold, the main manuscript reports feature stability as selection frequency across LOOCV folds.

Spectral region	Preprocessing pipeline	No. selected features	Selected wavenumbers (approx., cm^{-1})
600–1900 cm^{-1}	2nd derivative + normalization	9	≈713, ≈732, ≈767, ≈830, ≈911, ≈923, ≈929, ≈949, and ≈1283 cm^{-1}
2800–3400 cm^{-1}	2nd derivative + normalization	7	≈2832, ≈2883, ≈2903, ≈3042, ≈3047, ≈3054, and ≈3188 cm^{-1}
